# Response to targeted radionuclide therapy with [^131^I]MIBG AND [^177^Lu]Lu-DOTA-TATE according to adrenal vs. extra-adrenal primary location in metastatic paragangliomas and pheochromocytomas: A systematic review

**DOI:** 10.3389/fendo.2022.957172

**Published:** 2022-10-20

**Authors:** Stefan Prado-Wohlwend, María Isabel del Olmo-García, Pilar Bello-Arques, Juan Francisco Merino-Torres

**Affiliations:** ^1^ Nuclear Medicine Department, University and Polytechnic Hospital La Fe, Valencia, Spain; ^2^ Endocrinology and Nutrition Department, University and Polytechnic Hospital La Fe, Valencia, Spain; ^3^ Medicine Department, Universitat de València, Valencia, Spain

**Keywords:** paraganglioma, 131I-MIBG therapy, PRRT (peptide receptor radionuclide therapy), theragnostic agents, pheochromocytoma

## Abstract

**Purpose:**

Targeted radionuclide therapy (TRT) with [^131^I]MIBG and [^177^Lu]Lu-DOTA-TATE is an alternative treatment to the classic schemes in slow progressive metastatic/inoperable paraganglioma (PGL) and pheochromocytoma (PHEO). There is no consensus on which treatment to administer and/or the best sequence in patients who are candidates for both therapies. To clarify these questions, this systematic review assesses the prognostic value of [^131^I]MIBG and ^[177^Lu]Lu-DOTA-TATE (PRRT-Lu) treatments in terms of progression-free survival (PFS) both globally and considering the primary location.

**Methods:**

This review was developed according to the PRISMA Statement with 27 final studies (608 patients). Patient characteristics, treatment procedure, and follow-up criteria were evaluated. In addition, a Bayesian linear regression model weighted according to its sample size and an alternative model, which also included an interaction between the treatment and the proportion of PHEOs, were carried out, adjusted by a Student’s *t* distribution.

**Results:**

In linear regression models, [^131^I]MIBG overall PFS was, on average, 10 months lower when compared with PRRT-Lu. When considering the interaction between treatment responses and the proportion of PHEOs, PRRT-Lu showed remarkably better results in adrenal location. The PFS of PRRT-Lu was longer when the ratio of PHEOs increased, with a decrease in [^131^I]MIBG PFS by 1.9 months for each 10% increase in the proportion of PHEOs in the sample.

**Conclusion:**

Methodology, procedure, and PFS from the different studies are quite heterogeneous. PRRT-Lu showed better results globally and specifically in PHEOs. This fact opens the window to prospective trials comparing or sequencing [^131^I]MIBG and PRRT-Lu.

## Introduction

Paragangliomas (PGLs) and pheochromocytomas (PHEOs), altogether PGGLs, are uncommon neuroendocrine chromaffin tumors with an incidence of about 0.6 case per 100,000 persons per year. PHEOs are more common (80%–85%) and arise from the adrenal medulla, while PGLs are less frequent extra-adrenal tumors (15%–20%) derived from sympathetic or parasympathetic ganglia (1).

PGGLs are the most heritable of all human tumors, but paradoxically there is a wide genetic heterogeneity, with almost 32 genes described until the present and related to this disease (2). At least one-third of these patients carry a germline mutation, sorted in three different clusters: pseudohypoxia (cluster 1), kinase signaling (cluster 2), and Wnt signaling (cluster 3). The cluster will determine the primary locations, special molecular features, risk of metastatic disease, and avidity for nuclear medicine radiotracers (3–5). 

It is difficult to predict the individual tumoral behavior of PGGLs. Half of them are slow-growing, but the other half behave aggressively, that the term benign or malignant PGGL has been replaced by a risk stratification approach (6).

In limited focal disease, the surgical treatment is the only curative approach. In metastatic disease, the overall 5-year survival is 60%–70%, and the decision as to whether systemic treatment is appropriate is based on multiple factors. The “wait-and-see” strategy may be an option in those patients without any evidence of disease progression, but in symptomatic, progressive, or high-tumor-burden disease, a systemic treatment should be administered (7).

Among the systemic choices, chemotherapy with cyclophosphamide-vincristine-dacarbazine and temozolomide is the classic primary option in patients with quick progression. Targeted therapies such as anti-angiogenic tyrosine-kinase inhibitors, everolimus, and alpha-interferon have been tested in different disease stages and progression indexes, but many questions still remain unsolved.

Among the few therapeutic options, targeted radionuclide therapies (TRTs) are promising and generally well-tolerated treatments that can reach an adequate symptomatic control with little interference in a patient’s life. There are two options within TRT: ^131^I-metaiodobenzylguanidine ([^131^I]MIBG) and [^177^Lu]Lu-DOTA-TATE, also called peptide receptor radionuclide therapy (PRRT-Lu). Both treatments are alternatives to the classic schemes, at least in slow progressive patients, and in many cases, radiopharmaceuticals and even a sequential treatment with them may be appropriate in the same patient. The first step to plan a TRT is to evaluate the therapeutic targets with specific nuclear medicine scans: [^123^I]MIBG to select the therapy with [^131^I]MIBG and somatostatin receptor (SSTR) scans (SSTR SPECT/CT and [^68^Ga]Ga-DOTA-TOC PET/CT) to select PRRT. If the tumor expresses only one target, the decision is easy. However, overexpression of both targets has been described in up to 50% of patients, and it is in those cases where a question arises: which treatment is the most appropriate and/or in what sequence? (8, 9). In other cases, the tumors do not adequately express any of the specific targets and will not be candidates for TRT. The characteristics of the TRT modalities are summarized in **Table 1**.

**Table 1:** TRT modality characteristics.

Targeted radiolabeled therapy	^131^I-MIBG (Low-specific-activity)	^177^Lu-DOTATATE
Target	Norepinephrine transporter (principally)	Somatostatin receptors (especially subtype 2)
Phenotypic imaging scans	^123^I-MIBG	^111^In-Pentetreotide/^99m^Tc-HYNIC-TOC SPECT/CT, ^68^Ga-DOTATOC/NOC/TATE PET/CT
Average beta emission	0.192 Mev	0.134 MeV
Mean tissue range	0.5mm	0.23 mm
Storage	Neuroendocrine vesicles	Lysosomes
FDA approval	Metastatic/inoperable PGGL	Well-differentiated metastatic/inoperable GEP-NETs

Within the PRRT modality, there are two radiopharmaceuticals: [^177^Lu]Lu-DOTA-TATE (PRRT-Lu) and [^90^Y]Y-DOTA-TOC (PRRT-Y). The most commonly used agent in PGGLs is PRRT-Lu, and therefore in this review we will focus on it. PRRT-Y has a better profile for larger lesions, while PRRT-Lu has more favorable characteristics to treat small-volume metastatic lesions, such as those present in PGGLs. PRRT-Lu was approved in the treatment of well-differentiated metastatic/inoperable gastroenteropancreatic neuroendocrine tumors, but in metastatic/inoperable PGGL, its use is limited to clinical trials or compassionate use in many countries, so that the published data are limited to small uncontrolled retrospective studies (10, 11).

There are two sorts of [^131^I]MIBG radiopharmaceuticals: low-specific-activity or conventional [^131^I]MIBG (LSA [^131^I]MIBG), which is available worldwide and has been used for decades, and high-specific-activity [^131^I]MIBG (HAS [^131^I]MIBG) approved by the FDA in 2018 but not yet available in many countries. In LSA [^131^I]MIBG, which is used up to date, more than 99% of MIBG molecules are not labeled with ^131^I (cold MIBG). In HAS [^131^I]MIBG, the labeling process is 100–200 times more efficient, reducing the side effects and the competitiveness with the cold MIBG and reaching probably better results than LSA [^131^I]MIBG, but several studies will be needed to support it when use of this new therapeutic agent becomes widespread (12).

There is not yet any consensus or therapeutic guide on which TRT radiopharmaceutical should be used, and their place in the therapeutic algorithm is unclear. The ESMO-EURACAN clinical guidelines for metastatic PGGLs published in 2020 recommended an individualized management approach in case of disease progression, including TRT among chemotherapy, local therapies, or additional treatments (13).

The aim of this systematic review in metastatic PGGLs is to assess patient characteristics, treatment procedure, follow-up, and differences in the global response and as a function of the primary location of [^131^I]MIBG and [^177^Lu]Lu-DOTA-TATE in terms of progression-free survival (PFS). These results could facilitate the selection of the best option in those patients who can be treated with both therapies or in whom a sequential treatment is proposed.

## Material and methods

**Figure 1:**
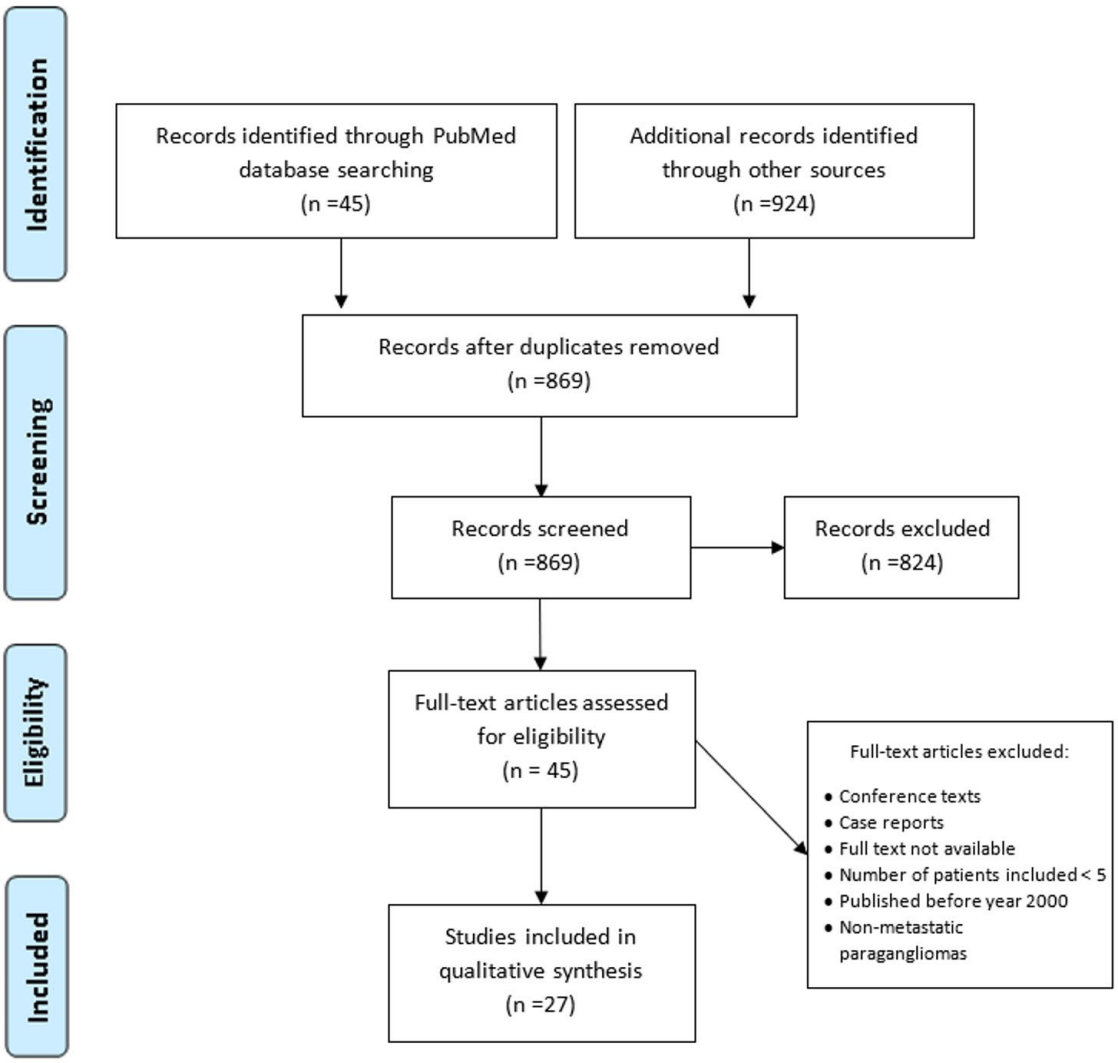
Flow chart of the search strategy and inclusion/exclusion process.

This systematic review was developed according to the Preferred Reporting Items for Systematic Reviews and Meta-Analyses (PRISMA) statement and conducted using MEDLINE (accessed from PubMed), Google scholar, and ClinicalTrials.gov. The search strategy was based on the Population, Intervention, Comparator, Outcome (PICO) framework and was designed to find studies and reviews including a combination of medical subject headings (MeSH) and non-MeSH keywords related to treatment with radiopharmaceuticals ([^177^Lu]Lu-DOTA-TATE and [^131^I]MIBG) in patients with metastatic paraganglioma and/or pheochromocytoma: “Peptide Receptor Radionuclide Therapy” or “131I-metaiodobenzylguanidine” and “metastatic paraganglioma and/or pheochromocytoma”. Papers referring exclusively to HAS [^131^I]MIBG or PRRT-Y were excluded. The total number of final studies included in the qualitative synthesis was 27 **(** **Figure 1** **)**.

Patient characteristics, treatment procedure, and administered activity were reviewed. Response to PRRT-Lu and [^131^I]MIBG was assessed through PFS. PFS was assessed from the time of treatment performed on disease progression, both overall and based on the primary location of the tumor. The evaluation criteria mentioned in the reports were biochemical and radiological according to Response Evaluation Criteria in Solid Tumors (RECIST) and through clinical follow-up.

### Statistical analysis

Data were summarized using mean (standard deviation) and median (first, third quartile) in the case of numerical variables, whereas relative and absolute frequencies were applied in the case of categorical variables. A descriptive study of patient variables and a calculation of the unweighted PFS means were performed. To assess differences in PFS between both treatments, a Bayesian linear regression model was adjusted where each study was weighted according to its sample size, including treatment, age, year of the study, and proportion of PHEOs as explicative variables. Additionally, an alternative model was adjusted where the interaction between the treatment and the proportion of PHEOs was also included. The model was fitted using a Student’s *t* distribution for likelihood instead of Gaussian to deal with outliers in the data and to make the model more robust to them. In both models, 95% credibility intervals were estimated for each parameter. The effect of the different explicative variables was also represented in conditional effect plots. All statistical analyses were performed using R (version 4.0.3) and R package brms (version 2.14.4).

## Results

This systematic review included 27 studies, 4 of them prospective and 23 retrospective **(** **Table 2** **)**. Nine studies were carried out with PRRT-Lu, 17 with [^131^I]MIBG, and one study included both modalities.

**Table 2:** Studies included in this systematic review.

TYPE	Year of publication	TRT	Total patients	Number of PGGL patients	Primary location	PFS (months)	PFS (PGL)	PFS (PHEO)	Reference
Retrospective	2017	177Lu-DOTATATE	20	20	PHEO 8 PGL 12	39	ns*	ns	(Kong et al., 2017) (14)
Retrospective	2019	177Lu-DOTATATE	30	30	PHEO 3 PGL 27	30	31	10	(Zandee et al., 2019) (15)
Retrospective	2006	177Lu-DOTATATE	22	12	PHEO 1 PGL 12	22	ns	ns	(Van Essen et al., 2006) (16)
Retrospective	2016	177Lu-DOTATATE	5	5	PHEO 0 PGL 5	17	ns	ns	(Pinato et al., 2016) (17)
Prospective	2017	177Lu-DOTATATE	143	5	PHEO 1 PGL 4	ns	ns	ns	Hamditibar (et al. 2017) (18)
Prospective	2018	177Lu-DOTATATE	200	5	PHEO 2 PGL 3	14	ns	ns	(Garske-Roman et al., 2018) (19)
Retrospective	2018	177Lu-DOTATATE	186	12	ns	31,4	ns	ns	(Demirci et al., 2018) (20)
Retrospective	2019	177Lu-DOTATATE	25	25	PHEO 0 PGL 25	32	ns	ns	(Yadav et al., 2019) (21)
Retrospective	2017	177Lu-DOTATATE	22	2	PHEO 0 PGL 2	38,5	38,5	ns	(Nastos et al., 2017) ** (22)
Retrospective	2020	177Lu-DOTATATE	15	15	PHEO 5 PGL 10	Not reached	Not reached	14	(Jaiswal et al., 2017) (23)
Retrospective	2017	131I-MIBG	22	13	PHEO 6 PGL 7	20,6	14,4	ns	(Nastos et al., 2017) **
Retrospective	2001	131I-MIBG	6	6	PHEO 5 PGL 1	12	ns	ns	(Hartley et al., 2001) (24)
Retrospective	2001	131I-MIBG	37	15	PHEO 8 PGL 7	ns	ns	ns	(Mukherjee et al., 2001) (25)
Retrospective	2003	131I-MIBG	25	6	PHEO 0 PGL 6	25,8	ns	ns	(Bomanji et al., 2003) (26)
Retrospective	2003	131I-MIBG	33	33	PHEO 22 PGL 11	ns	ns	ns	(Safford et al., 2003) (27)
Retrospective	2007	131I-MIBG	19	19	PHEO 13 PGL 6	28,5	ns	ns	(Gedik et al., 2008) (28)
Prospective	2009	131I-MIBG	50	50	PHEO 15 PGL 35	ns	ns	ns	(Gonias et al., 2009) (29)
Retrospective	2010	131I-MIBG	10	10	PHEO 7 PGL 3	17,5	ns	ns	(Shilkrut et al., 2010) (30)
Retrospective	2011	131I-MIBG	32	12	PHEO 8 PGL 4	29	ns	ns	(Rachh et al., 2011) (31)
Retrospective	2011	131I-MIBG	16	6	PHEO 6 PGL 0	48	ns	48	(Szalat et al., 2011) (32)
Retrospective	2012	131I-MIBG	17	5	PHEO 0 PGL 5	11,26	ns	ns	(Fishbein et al., 2012) (33)
Retrospective	2014	131I-MIBG	70	48	PHEO 37 PGL 11	ns	ns	ns	(Yoshinaga et al., 2014) (34)
Prospective	2006	131I-MIBG	30	30	PHEO 11 PGL 19	ns	ns	ns	(Fitzgerald et al., 2006) (35)
Retrospective	2013	131I-MIBG	26	26	PHEO 8 PGL 18	ns	ns	ns	(Wakabayashi et al.2019) (36)
Retrospective	2010	131I-MIBG	12	12	PHEO 4 PGL 8	30	ns	ns	Castellani group 1 before 2001. Low activity (Castellani et al., 2010) (37)
Retrospective	2010	131I-MIBG	16	16	PHEO 11 PGL 5	24,92	ns	ns	Castellani group 2 after 2001. Intermediate activity (Castellani et al., 2010)
Retrospective	2003	131I-MIBG	12	12	PHEO 6 PGL 6	32,25	ns	ns	(Rose et al., 2003) (38)
Retrospective	2015	131I-MIBG	22	22	PHEO 10 PGL 12	ns	ns	ns	(Rutherford et al., 2015) (39)
Retrospective	2020	131I-MIBG	125	125	PHEO 73 PGL 52	ns	ns	ns	(Thorpe et al., 2020) (40)

*Nonspecified. **Study including patients treated with both therapeutic modalities.

Only four reports provided PFS based on the adrenal or extra-adrenal location. In relation to the line in the treatment scheme, few articles specified it. [^131^I]MIBG was preferably administered as the first systemic line (73.4%), whereas PRRT-Lu was administered first line in 39.7% and second line in 38.5%. Most studies did not report the genetic syndromes of the patients (especially in [^131^I]MIBG), so this variable could not be assessed. Regarding the response evaluation criteria, 10 articles evaluated biochemical criteria, 21 RECIST, and 10 clinical follow-up.

**Figure 2:**
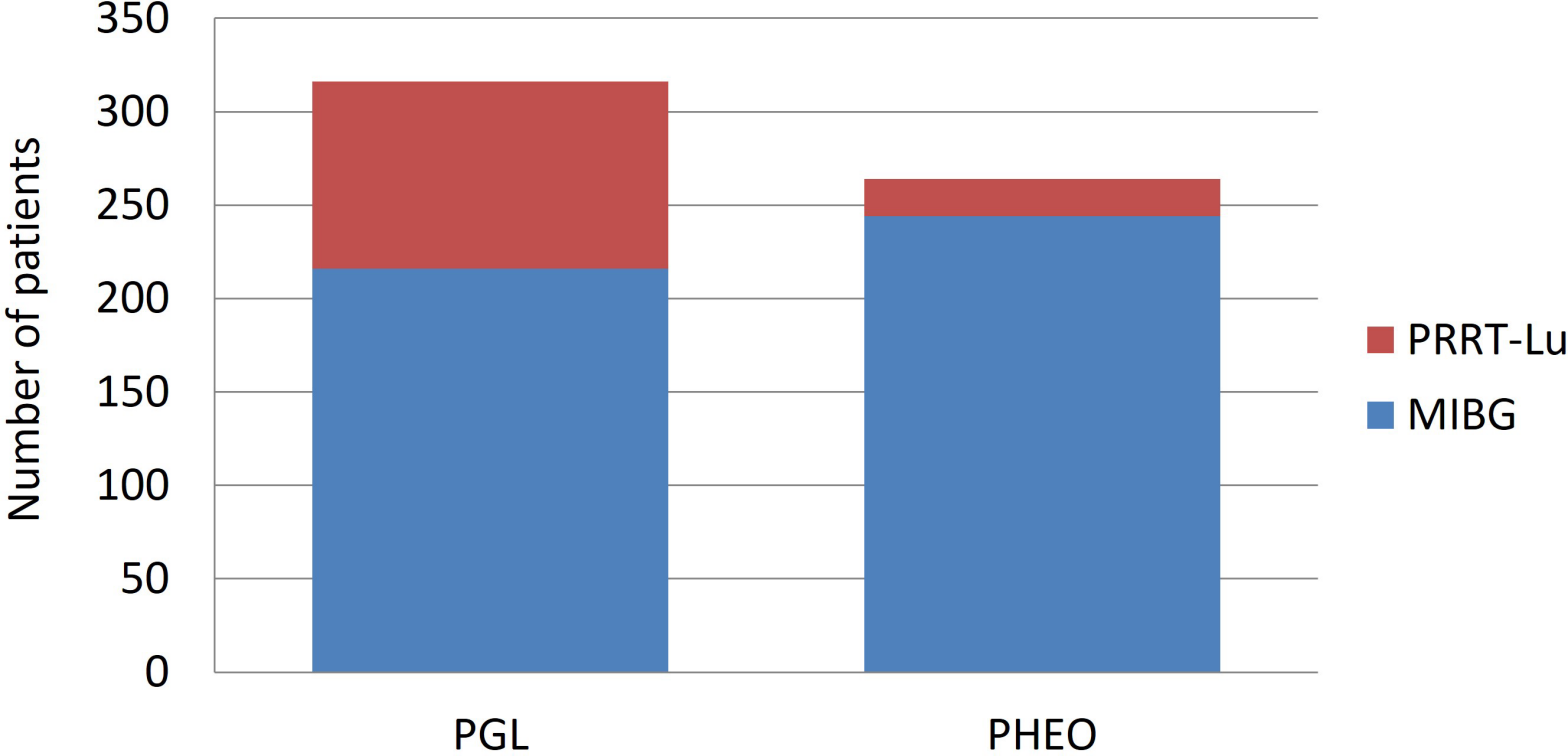
Distribution of the administered treatments according to the adrenal or extra-adrenal location of the tumor.

A total of 1248 patients were reported in the series, of which 608 were PGGL (264 PHEO and 316 PGL, and in the remaining 28 patients, the primary location was not specified) **(** **Table 3** **)**. The largest number of patients was treated with [^131^I]MIBG (76.6%). In the PRRT-Lu group, most of the patients were PGL (83.3%), whereas in the [^131^I]MIBG group, the numbers of PGL and PHEO were similar (46.9 vs. 53%). The vast majority of PHEOs were treated with [^131^I]MIBG (92.4%) **(** **Figure 2** **)**.

**Table 3:** Patient descriptive variables according to the treatment modality. In some variables, the data specified in the papers are scarce.

Treatment	Total patients PGGL	Primary PGL	Primary PHEO	Mean age (years)	Women/Men	First-line therapy	Second-line therapy	Third-line therapy	Fourth-line therapy and successive	Cumulative activity range (GBq)	Follow up (months)
**^131^I-MIBG**	466 (76.6%)	216 (68.3%)	244 (92.2%)	42.8 (32.5-52)	205/261	36	12	1	–	7.4–39.4	28.6
**PRRT-Lu**	142 (23.3%)	100 (31.6%)	20 (7.5%)	43.7 (30.9–51)	52/90	33	32	11	7	22–29.6	31.4

**Figure 3:**
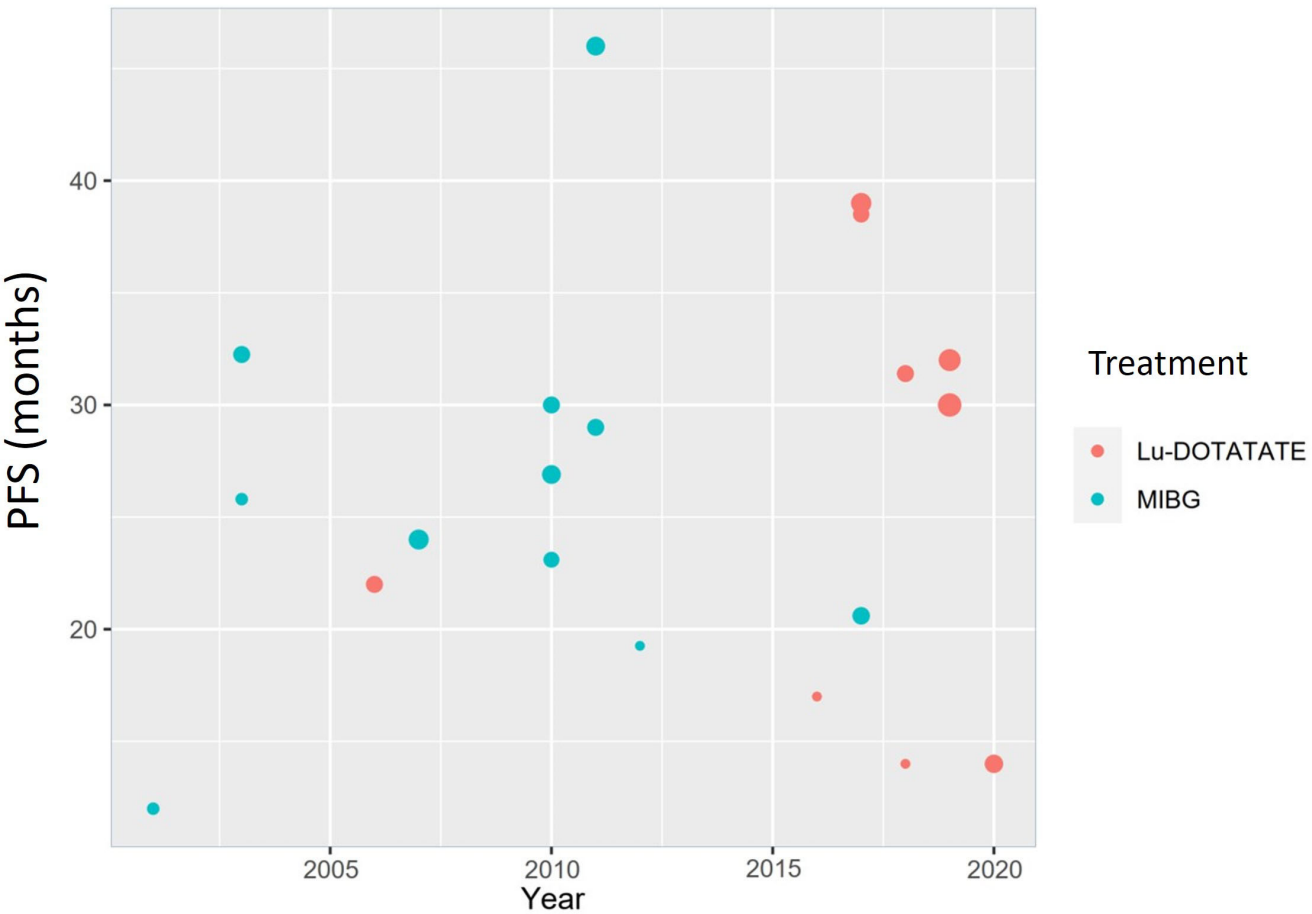
PFS estimates from the different studies between 2001 and 2020 with [^177^Lu]Lu-DOTA-TATE (red) and [^131^I]MIBG (blue). Dot sizes are proportional to the sample size of the study.

The PFS estimates from the reviewed studies show a very large spread (12–46 months), and there is also no clear pattern regarding years or treatment modality **(** **Figure 3** **)**. The unweighted mean of PFS was 25.43 months for [^131^I]MIBG and 29.55 for PRRT-Lu, with no statistical significance (p: 0.787) (95% CI [-5.9, 14,1]). The PFS mean based on the primary location could not be calculated due to the lack of specific data in the reports.

In the regression model weighted for each report according to the corresponding sample sizes, [^131^I]MIBG showed lower PFS when compared with PRRT-Lu. Specifically, the [^131^I]MIBG yielded, on average, 10 months lower PFS than PRRT-Lu (95% CI [-11.7, -8.5]) **(** **Table 4** **)**.

**Table 4:** Regression model comparing PFS between [^131^I]MIBG and [^177^Lu]Lu-DOTA-TATE treatments.

Variables	Estimate	Std error	Lower 95	Upper 95
Age	-0.327	0.031	-0.39	-0.27
Year	-0.37	0.051	-0.473	-0.272
MIBG treatment	-10.066	0.824	-11.722	-8.504
Prop PHEO	-2.699	1.625	-5.496	0.793

The variables under study were age, year of publication, [^131^I]MIBG treatment, and proportion of PHEOs.

As only four reports provided PFS based on the primary location and the previous statistical model did not consider that the treatments do not affect adrenal and extra-adrenal primary location equally, we adjusted a new Bayesian linear regression model considering a possible interaction between treatment responses and the proportion of PHEOs. This new model revealed very different results, and the difference between [^131^I]MIBG and PRRT-Lu was dependent on the proportion of PHEOs in each study. PRRT-Lu increased the PFS in PHEOs comparatively with [^131^I]MIBG. [^131^I]MIBG showed larger PFS than PRRT-Lu when the proportion of PHEOs was low, and PFS was significantly longer with PRRT-Lu when the proportion of PHEOs increased **(** **Table 5** **)**. Specifically, with no PHEOs, the mean difference was 5.18 months (95% CI [4.16, 6.06]) in favor of [^131^I]MIBG, and it decreased by 1.9 months (95% CI [-2.01, -1.78]) for each 10% increase in the proportion of PHEOs in the sample.

**Table 5:** Regression model comparing PFS between [^131^I]MIBG and [^177^Lu]Lu-DOTA-TATE considering that the treatments do not affect equally the adrenal and extra-adrenal primary location.

Variables	Estimate	Std Error	Lower 95	Upper 95
Age	-0.447	0.008	-0.463	-0.429
Year	-0.285	0.012	-0.311	-0.266
MIBG treatment	5.181	0.484	4.157	6.063
Prop PHEO	14.009	0.559	12.824	15.018
MIBG treatment/prop PHEO	-19.046	0.575	-20.126	-17.845

The variables under study were age, year of publication, treatment with [^131^I]MIBG, proportion of PHEO, and proportion of PHEO treated with [^131^I]MIBG.

**Figure 4:**
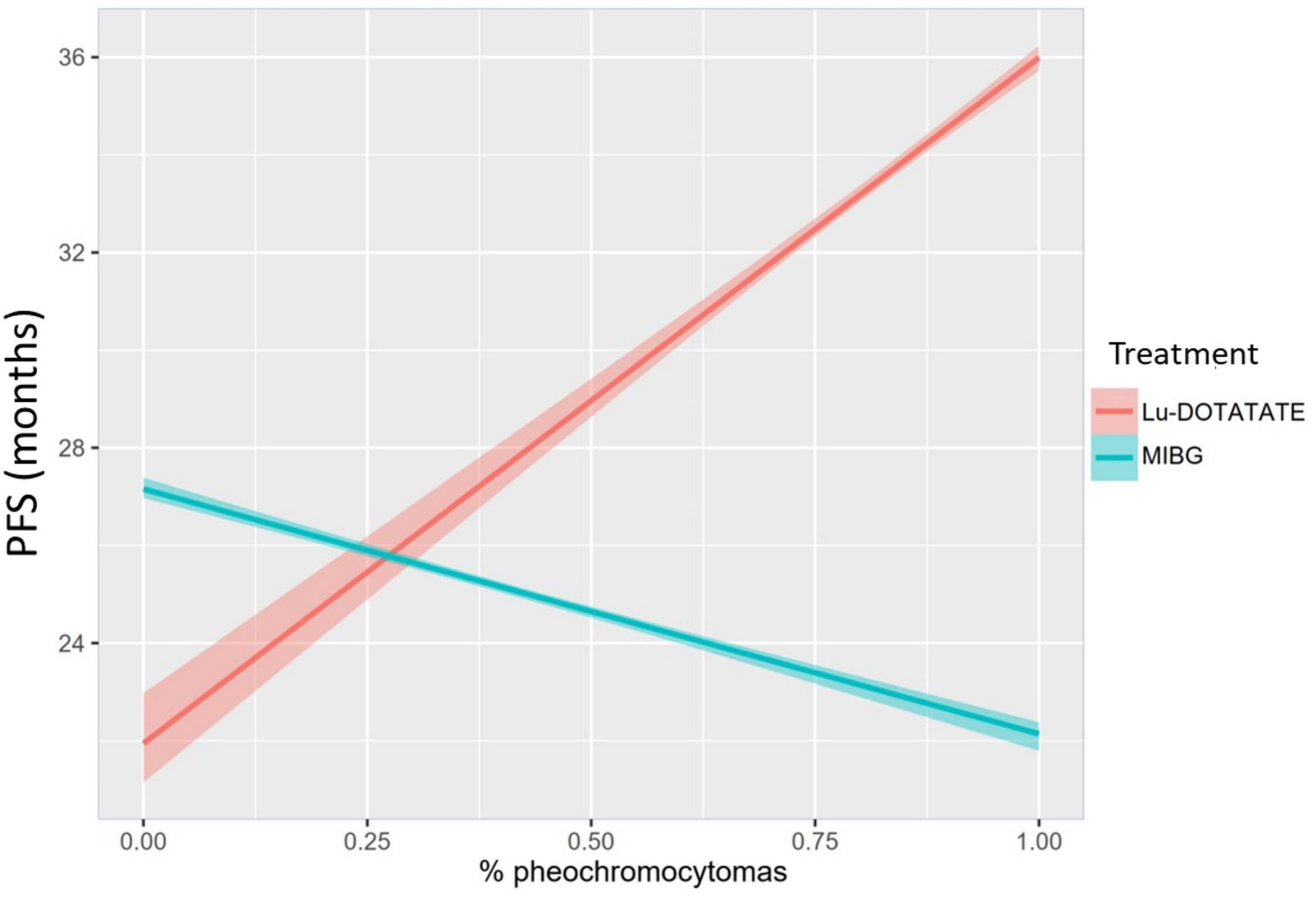
Conditional effects plot depicting the interaction between treatment and proportion of PHEOs in the sample regarding the estimated PFS in months.

To aid in the interpretation of the interaction model, we also estimated a conditional effects plot, describing the relationship between both treatments, the proportion of PHEOs in the sample, and the estimated PFS **(** **Figure 4** **)**.

## Discussion

PGGLs have heterogeneous presentation and prognosis. This fact, added to the lack of current guidelines and controlled studies, leads physicians to a pragmatic personalized approach based on the experts’ consensus and a benefits–risk balance.

In the [^131^I]MIBG series, there is large heterogeneity in the published average cumulative dose (7,4-39,4 GBq), the number of administered cycles (1–12), and PFS (12–32.2 months).

Most of the [^131^I]MIBG series are retrospective case reports, and there is only one prospective study reported by Gonias et al. (29). This one involved 50 patients with administered activities ranging from 18.2 to 118 GBq, reaching responses in 22% of the cases but at the expense of significant toxicities.

In [^131^I]MIBG, the balance between the benefits and comorbidities of a high-activity treatment remains unclear. Castellani et al. (37) compared two groups of patients using an intermediate vs. low cumulative dose (39.4 vs. 24.1 GBq) and concluded that the most important result using higher activity without reaching myeloablative levels was the shortened time to achieve a significant response. Low-dose treatment results reported by Shilkrut et al. (30) (one to four cycles) with 11.6 GBq obtained a PFS of 17.5 months, and those reported by Rachh et al. (31) (one to five cycles) with 11.8 GBq reached a PFS of 29 months. At the other end, Rose et al. (38) using high therapeutic doses (29.6 GBq) observed a 32.2-month PFS.

Studies with [^131^I]MIBG that specifically classify their results according to adrenal or extra-adrenal primary location are lacking. Safford et al. (27) observed a trend to decreased survival in those patients with metastatic PGL vs. PHEO (1.8 vs. 4.7 years, respectively), whereas they did not get a statistically significant difference.

As opposed to [^131^I]MIBG, PRRT-Lu published series are quite homogeneous in the cumulative dose that ranges between 22 and 29.6 GBq. However, we found more variability in PFS, ranging between 14 and 39 months. There are only three prospective studies, two of them with five PGGLs and the third one with 20 PGGLs published by Kong et al. (14)Hamiditabar et al. (18), and Garske-RomáVerify that all the equations and special characters are displayed correctly.n et al. (19), respectively. Among the few cases in which the papers specified the therapeutic line, and in contrast to [^131^I]MIBG, most of the PPRT-Lu treatments were not administered as the first line, probably because PRRT-Lu is a more recent treatment, and it is limited to compassionate use in many countries.

The largest PRRT-Lu series, involving 30 patients and published by Zandee et al. (15), observed an overall PFS of 30 months and divided the results according to the adrenal vs. extra-adrenal location. They obtained a 30-month PFS in 10 patients with parasympathetic PGL, 13-month PFS in the sympathetic PGLs, and 8–10–14 months in the three PHEO patients.

When considering TRT, the extension and intensity uptake of the targets should be evaluated both on [^123^I]MIBG and SSTR scans and compared with the whole tumor burden noted on CT/MRI and/or ^18^F-FDG PET/CT. Less than 50% of metastatic PGGLs display [^123^I]MIBG avidity. On the other hand, SSTRs are overexpressed in more than 80% of neuroendocrine tumors. In many cases, differences between them are obvious (normally due to the lack of uptake in one of them). But if both scans reveal a similar uptake and extent of disease, the choice becomes complicated. There is only one study comparing the therapeutic outcomes of both TRT modalities published by Nastos et al. (22). They used [^131^I]MIBG or a combination of [^131^I]MIBG and PRRT (most PRRT-Y) and observed that patients treated with PRRT had an increased PFS and response to treatment compared with [^131^I]MIBG (p < 0.05) but with no significant difference in overall survival. When comparing only PGL patients, Nastos et al. (22) observed that response to treatment, overall survival, and PFS were significantly higher in the PRRT group. In this study, the effectiveness of PRRT in PHEOs could not be assessed because, of the seven patients with this primary location, only one was treated with PRRT.

Combining both [^131^I]MIBG and PRRT could be a future alternative especially in those patients with aggressive disease. In this line, Bushnell et al. (41) published a phase 1 clinical trial combining treatment with PRRT-Y and [^131^I]MIBG with pretreatment tumor dosimetry in three patients with neuroendocrine tumors, with an adequate safety margin (41).

**Figure 5:**
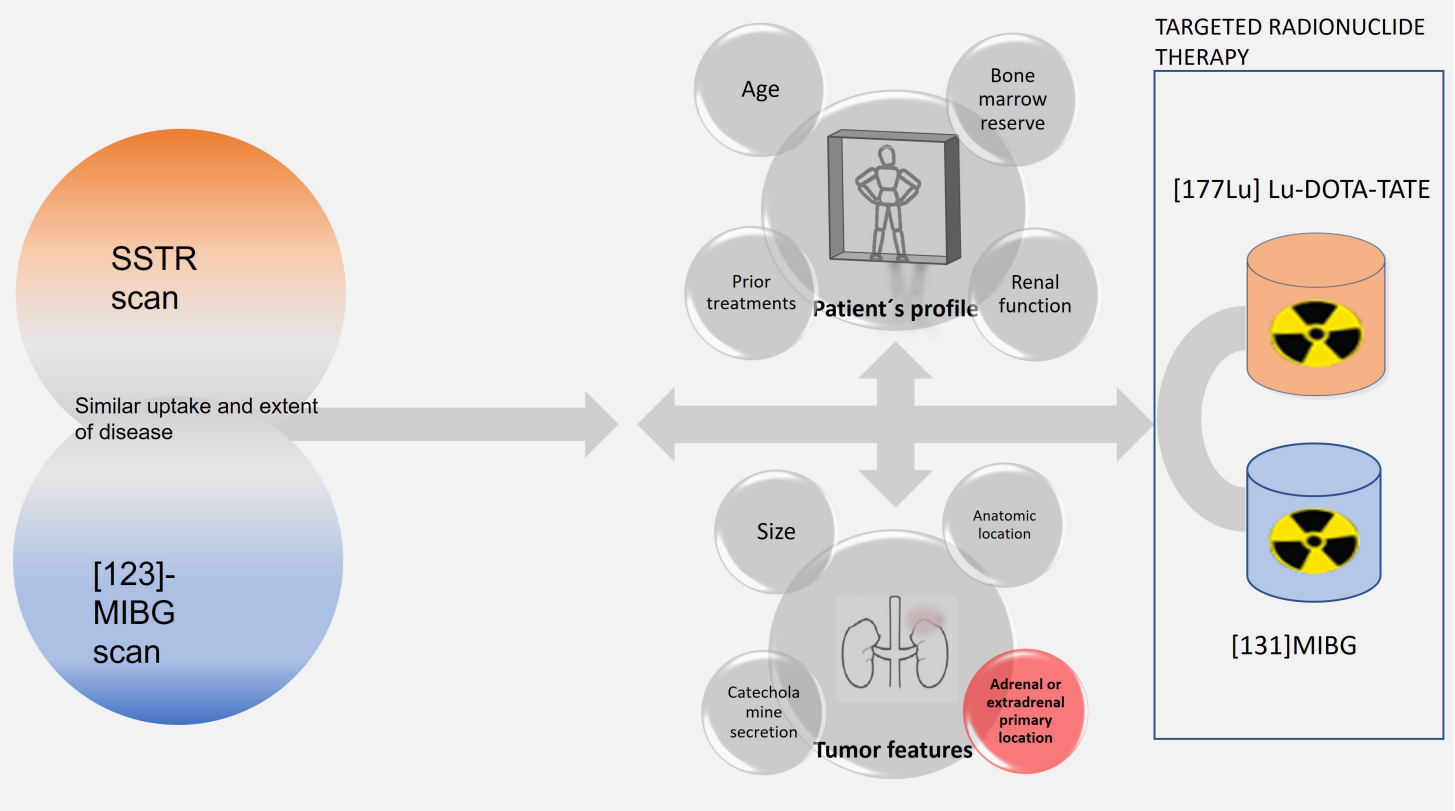
Flow chart of functional imaging findings, patient profile, and tumor features involved in the selection of TRT.

There are no large prospective studies comparing [^131^I]MIBG and PPRT-Lu, if both are available and similarly suitable. In the Jha etal. report (8), a group of international experts facing this obstacle recommends considering, in addition to nuclear medicine scans, the patient’s profile and the tumor characteristics and prognostic features to guide the decision. If mismatched uptakes are found in nuclear medicine scans, these authors suggest a combined sequence of both TRTs to cover all the lesions. Unfortunately, there are still not enough prognostic data available, based on these variables, to support which TRT to choose **(** **Figure 5** **)**.

We found several limitations in this review. Firstly, the samples of both treatments were not homogeneous, and, in 28 patients, the primary location of the tumor was not identified, so the means obtained could not be fully compared. In most of the reports, PGGLs were a small number of patients included in larger neuroendocrine tumor series. The majority of the studies did not provide PFS according to the location, and for this reason, linear regression statistical models had to be carried out. Within PHEOs, only 20 patients were treated with PRRT-Lu. However, they were extracted from five different reports, so it is unlikely that the results were due to the bias of a single study. In neuroendocrine tumors, a correlation has been observed between the high uptake of lesions in SSTR scans and the density of SSTR2 expression in biopsies. A higher SSTR density may imply a better response to PRRT, so patients who strongly express this target are preferentially selected for PRRT rather than for [^131^I]MIBG. Given that patients with high SSTR expression are expected to have a better response to PRRT, this fact may imply a patient selection bias due to falsely improving PRRT results (42). Additionally, in the [^131^I]MIBG group, we observed significant heterogeneity in the administered cumulative activities, although it showed a low impact on the PFS, and the reviewed reports did not distinguish whether all patients were candidates for both treatments. Most of the reviewed articles did not report to which cluster the genetic syndromes belonged, so a correlation could not be done between response to treatment and genetic status.

Despite these limitations, we observed PFS differences globally and according to the primary location and the therapeutic modality administered. The unweighted means and the statistical model weighted to represent the sample size showed greater PFS in the PRRT-Lu group. The model weighted to the proportion of PHEOs showed that the adrenal location responded better to treatment with PRRT-Lu. In patients expressing both targets, this fact could support the selection of PRRT-Lu as a first option, especially in PHEOs, and if a TRT sequence with both therapies is considered, the first one to be administered.

## Conclusion

Methodology, procedure, and PFS from the different reviewed studies about TRT in metastatic/inoperable PGGLs are quite heterogeneous. PRRT-Lu seems to have overall better results, particularly in PHEOs, but this question should probably be reassessed shortly with the arrival of HAS [^131^I]MIBG. Although the reports present several limitations, our results make it clear that tumor primary location is a factor to consider in therapy planning.

These results should open the window to prospective trials comparing or sequencing [^131^I]MIBG and ^177^Lu-DOTATATE. Studies aimed at the evaluation of both treatments are necessary, with special attention to the primary location and the line of therapy, reaching similar administered cumulative activities and correlating them with the genetic cluster within each group.

## Data availability statement

The original contributions presented in the study are included in the article/Supplementary Material. Further inquiries can be directed to the corresponding author.

## Author contributions

SP-W and MO-G reviewed the articles and conducted the study. PB-A and JM-T formally and scientifically reviewed the manuscript. All authors contributed to the article and approved the submitted version.

## Funding

Funding for conducting the review has been received from the La Fe Hospital Research Foundation.

## Conflict of interest

The authors declare that the research was conducted in the absence of any commercial or financial relationships that could be construed as a potential conflict of interest.

## Publisher’s note

All claims expressed in this article are solely those of the authors and do not necessarily represent those of their affiliated organizations, or those of the publisher, the editors and the reviewers. Any product that may be evaluated in this article, or claim that may be made by its manufacturer, is not guaranteed or endorsed by the publisher.
